# Simulation Study of Surface Transfer Doping of Hydrogenated Diamond by MoO_3_ and V_2_O_5_ Metal Oxides

**DOI:** 10.3390/mi11040433

**Published:** 2020-04-20

**Authors:** Joseph McGhee, Vihar P. Georgiev

**Affiliations:** Device Modelling Group, University of Glasgow, Glasgow G128LT, Scotland, UK; j.mcghee.2@research.gla.ac.uk

**Keywords:** surface transfer doping, 2D hole gas (2DHG), diamond, MoO_3_, V_2_O_5_

## Abstract

In this work, we investigate the surface transfer doping process that is induced between hydrogen-terminated (100) diamond and the metal oxides, MoO_3_ and V_2_O_5_, through simulation using a semi-empirical Density Functional Theory (DFT) method. DFT was used to calculate the band structure and charge transfer process between these oxide materials and hydrogen terminated diamond. Analysis of the band structures, density of states, Mulliken charges, adsorption energies and position of the Valence Band Minima (VBM) and Conduction Band Minima (CBM) energy levels shows that both oxides act as electron acceptors and inject holes into the diamond structure. Hence, those metal oxides can be described as p-type doping materials for the diamond. Additionally, our work suggests that by depositing appropriate metal oxides in an oxygen rich atmosphere or using metal oxides with high stochiometric ration between oxygen and metal atoms could lead to an increase of the charge transfer between the diamond and oxide, leading to enhanced surface transfer doping.

## 1. Introduction

Diamond has many electronic applications, such as microwave electronic devices [1], bipolar junction transistor [2], and Schottky diodes [3]. However, one of the most promising areas for diamond industrial application is high-performance field effect transistors (FETs) in the production of high frequency and high-power electronic devices [4]. Its properties potentially enable devices that are beyond the scope of current systems in terms of operating frequency, power handling capacity, operating voltage, thermal robustness, and operating environment. This is due to the fact that the diamond has a wide band-gap of 5.5 eV, a thermal conductivity five times greater than 4H–SiC of 24 W/cm·K (for CVD diamond), a high breakdown field of 20 W·cm^−1^, and high hole and electron carrier velocities of 0.8 × 10^7^ cm/s and 2.0 × 10^7^ cm/s, respectfully; making it a superior new candidate for high frequency and high power devices [5,6,7,8,9]. However, the primary issue that has inhibited the application of diamond in the production of mature electronic devices is the lack of a suitably efficient and stable doping mechanism. It is well known that conventional substitutional doping is difficult to achieve in diamond in comparison to other semiconductor materials, such as Si and III-Vs [10]. It is possible to dope diamond with some atoms; however, boron being the most common and successful. Boron doping has its limitations; however, the hole mobility deteriorates as the doping concentration increases and when the doping concentration rises above 3.9 × 10^21^ cm^−3^ the diamond takes on semi metallic properties [11,12]. 

Surface Transfer Doping (STD) provides an alternative doping strategy that overcomes the intrinsic limitations that are associated with substitutional doping in diamond and, hence, presents a potential solution for the production of viable electronic devices [13,14]. For STD to occur in diamond, there are two main prerequisites: hydrogen termination of the diamond surface (H-diamond) and an electron accepting material in intimate contact with the H-diamond surface. The hydrogen termination gives the diamond surface a negative electron affinity, which facilitates the transfer of electrons from the diamond valance band to the surface electron accepting material, creating a quasi two-dimensional sub-surface hole gas (2DHG) in the diamond. 

**Figure 1:**
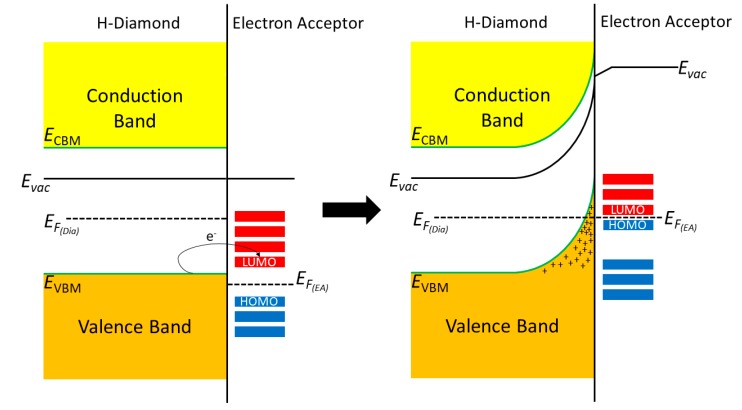
Band diagrams showing the interaction of hydrogen terminated diamond with a surface electron acceptor material.

Figure 1 shows the current model for the band bending mechanism in H-diamond when it comes into intimate contact with an electron acceptor material.

In the earliest developments of STD, the electron acceptor material was provided by molecules in the atmosphere that spontaneously adsorbed onto the surface of hydrogen-terminated diamond [15]. However, the lack of control over the adsorption of the atmospheric species, and the fact they are readily desorbed by elevated temperatures and fabrication processes, led to research into alternative electron acceptors that are controllable, more stable, and provide improved performance. The first experimental work to use alternative surface electron acceptors was by Strobel et al., who used high electron affinity molecules C_60_ and C_60_F_48_ in 2004 and 2005, respectively [16,17], with Edmonds et al. revisiting the use of C_60_F_48_ in 2012 [18]. In 2007, Qi et al. implemented the use of the strong electron withdrawing molecule F_4_-TCNQ as the surface acceptor to induce STD in H-diamond reporting areal hole density of 1.6 × 10^13^ cm^−2^ [19]. However, these alternative dopants were found to result in lower carrier concentrations than atmospheric adsorbates and were unstable at elevated temperatures, despite offering improved controllability of adsorbate on the H-diamond surface. High electron affinity metal oxides have been utilised as surface acceptor materials in order to improve the device stability and enhance the carrier concentration in the surface transfer doped H-diamond. Two of the metal oxides that have been shown in previous experimental studies to improve the performance and stability of STD in H-diamond are MoO_3_ and V_2_O_5_ [13,14,20,21,22,23,24,25,26,27]. 

**Figure 2:**
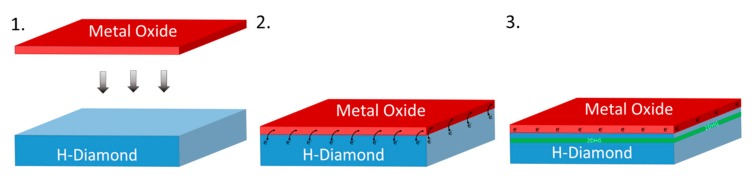
The creation of the two-dimensional sub-surface hole gas (2DHG) just below the diamond surface after interfaced with a suitable metal oxide.

Figure 2 presents a schematic diagram and the conceptual idea showing that when a metal oxide with suitably high electron affinity (~4.3 eV) is deposited on an H-diamond surface, electrons from just below the surface of the diamond will transfer to the metal oxide creating the 2DHG in the diamond. The higher the number of electrons extracted from the diamond substrate to the electron acceptor material, the higher the hole concertation of the 2DHG, which, in turn, reduces resistance and increases maximum current in electrical devices. Hence, it is desirable to maximise the charge transfer density from the diamond to the electron acceptor. Such an optimization of charge transfer is highly challenging when utilising randomly adsorbed species from ambient air on to the H-diamond surface. The species also prove to be unstable at elevated temperatures and offer poor device reliability. Therefore, the optimisation of the composition and structure of thin metal oxide films as alternative electron acceptor layers provides greater potential for engineering the STD process in diamond, and it can result in more robust thermal stability and device reliability.

When compared to the progress and publication of experimental work, there has been little theoretical investigation into the STD of H-diamond. In the past, ab-initio and Density Functional Theory (DFT) first principal studies have been carried out to model the types of interactions and transfer doping that occurs in the atmosphere by modelling molecules, such as HCl, NH_3_, H_2_O, NO_2_, NO, and O_3_ on H-diamond [28,29,30]. Until very recently, there has been a lack of theoretical investigation into metal oxide and H-diamond interfaces. Xiang et al. and Xing et al. investigated the STD between H-diamond and molecules of CrO_3_ and MoO_3_, respectively, while using DFT methods. The publications reported estimated carrier concentrations of 4.7 × 10^13^ cm^−2^ and 9.83 × 10^13^ cm^−1^ for MoO_3_ and CrO_3_ molecules, correspondingly [27,31].

In this work, we present DFT simulations of STD in H-diamond using automictically thin surface layers of both MoO_3_ and V_2_O_5_ in order to investigate these charge transfer processes and obtain detailed scientific understanding of the charge transfer phenomena in such kind of systems. The interfaces of (100) α-MoO_3_ and (100) V_2_O_5_ with 2 × 1-(100) H-diamond have been modelled and resultant STD analysed with a view to better understanding and optimising STD in the diamond. Although similar DFT studies have already been reported for CrO_3_ and MoO_3_ molecular clusters on diamond, this work models extended crystalline thin film structures that could better represent real experimental conditions.

## 2. Materials and Methods 

All of the calculations were carried out with Quantumwise Atomistix ToolKit (ATK) software (2017, Copenhagen, Denmark) while using the DFT method [32]. Generalised Gradient Approximation (GGA) exchange correlation was used for the geometry optimisations of all systems and obtain the total energies of the interfaced systems and the individual component parts i.e. H-diamond and the oxide layer in question. For all geometry optimisations, a force tolerance of 0.01 eV/Å was used. GGA-1/2 exchange correlation was used for all electronic structure calculations. The pure DFT method is well known for underestimating the bandgap of semiconductors. Therefore, the DFT-1/2 method was used to obtain a more accurate electronic description of the systems. The DFT-1/2 method is a semi-empirical approach that can overcome the error that local and semi-local exchange correlation density functionals inherently have when working with semiconductors and insulators. It works by correcting the self-interaction error of DFT by cancelling out the electron-hole self-interaction energy by defining an atomic self-energy potential [33]. However, the DFT-1/2 method is not suitable for calculating properties that depend on total energy; hence, we used GGA for the geometry optimisations and calculating adsorption energies. The Perdew, Burke, and Ernzerhof (PBE) functional was chosen for all calculations, because of the good match with experimental data (around 5.5 eV) regarding the value on the band gap in bulk diamond, as shown in Table 1. 

A Monkhorst-Pack scheme with an 8 × 8 × 1 k-point density (Å) mesh was used for the Brillouin zone integration. An iteration control tolerance of 0.0001 with a density cut off of 1 × 10^−6^ was used for all calculations with a medium basis set. The number of pseudo-atomic orbitals in a medium basis set is typically comparable to that of a double-zeta polarized (DZP) basis set [34]. The pseudopotential is SG15 and the density mesh cut-off is 185Ha, which gives high accuracy with a medium computational efficiency.

The H-diamond, MoO_3_, and V_2_O_5_ were created and geometry optimised individually by allowing all of the atoms to fully relax. After this optimisation, the H-diamond and the metal oxide structures were interfaced and a geometry optimisation process was performed by fixing the diamond structure while allowing for the oxides to move as a rigid body in all directions. The interface distances were corrected for counterpoise basis set superposition error and Van der Waals interactions. In this way, the computational cost is significantly reduced, as the surface atoms of the two structures were not fully relaxed independently. When the oxides were interfaced with the 2 × 1-(100) H-diamond, a strain of <1% was placed on the MoO_3_ and 1.02% was placed on the V_2_O_5_. 

## 3. Results

### 3.1. MoO_3_: H-Diamond Interface

**Figure 3:**
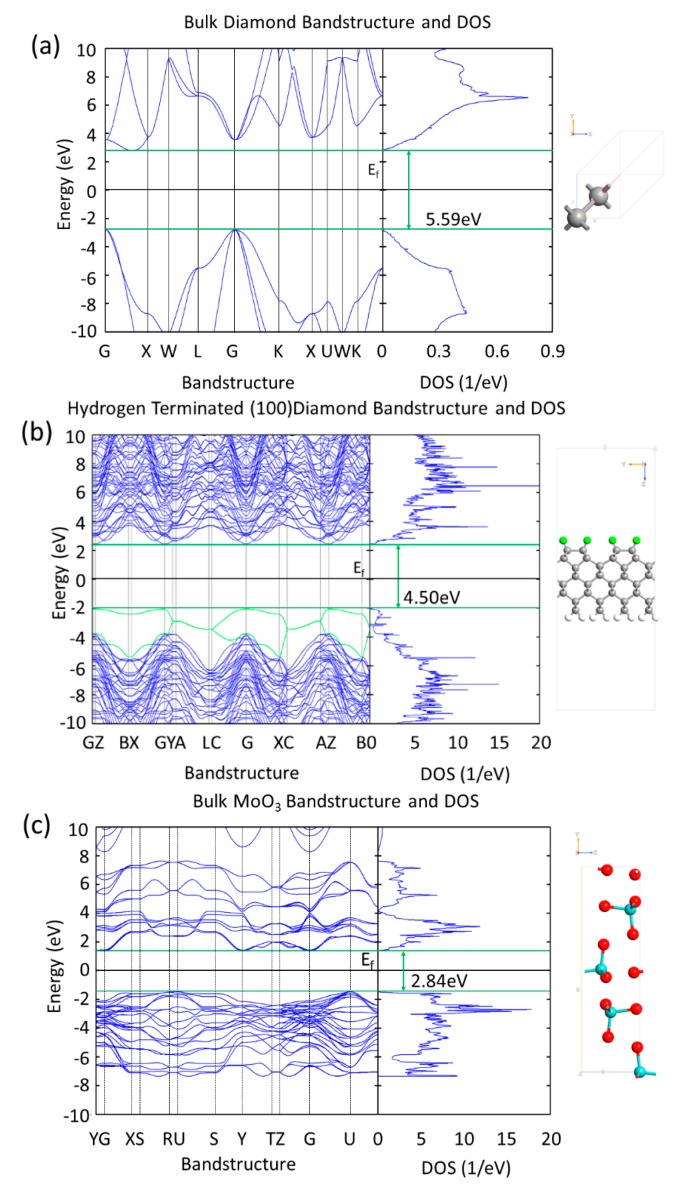
(**a**) Bandstructure and Density of States (DOS) of bulk diamond showing a band gap of 5.59 eV, (**b**) bandstructure and DOS of hydrogen terminated (100) diamond showing a reduced band gap of 4.50 eV due to the highlighted green hydrogen atoms, and (**c**) bandstructure and DOS of bulk MoO_3_ showing a band gap of 2.84 eV.

Figure 3 compares diamond, hydrogen terminated diamond and MoO_3_ band structures and Density of States (DOS) obtained from the unit cell and super cell DFT simulations. The lattice parameters of the three systems in Figure 3, are: (a) bulk diamond—face centered cubic a = 3.574 Å, (b) hydrogen terminated diamond—super cell lattice type a = 2.527 Å, b = 10.108 Å, c = 27Å (the unit cell for the hydrogen terminated 2 × 1 diamond is a = 2.527 Å, b = 5.054 Å and it is in agreement with the experimental values of 2.52 Å and 5.04 Å [35]), MoO_3_—simple orthorhombic a = 3.962 Å, b = 13.86 Å, c = 3.697 Å. The data that are presented in Figure 3a,b show that, when diamond is hydrogen terminated, the band gap is slightly reduced at the surface due to surface gap states at the valance band maximum (VBM) and conduction band minimum (VBM), because of the presence of hydrogen atoms—the most prominent additional bands from the C–H interaction at the VBM have been highlighted in green in Figure 3b. This is due to the bonding and antibonding C–H states at the VBM and CBM [36]. Although the band gap of the system has been reduced to 4.50 eV, which is shown in Figure 3b, the projected DOS plot for the carbon atoms in the bulk of the structure shows their band gap remains ~5.5 eV. The first two valance sub-bands in Figure 3b, which clearly have higher energies in comparison to the other sub-bands, arise from the interaction of the first layer of carbon with the adjacent hydrogen atoms. If these two sub-bands are ignored the band gap for diamond remains ~5.5 eV. 

The band gap of 2.84 eV that is obtained for MoO_3_ (Figure 3c) is comparable to reported experimental values of 3.2 eV and 2.8 eV for bulk and polycrystalline MoO_3_, respectfully, obtained by absorption spectra measurements [37]. Thermally evaporated MoO_3_ (as utilised in previous experimental work [20,26]) forms an amorphous film and, therefore, does not have a well-defined crystal structure. In our simulations, the MoO_3_ is built as a perfect crystal and it is most likely the reason that the band gap obtained from the DFT is underestimated in the comparison to the reported experimental values. 

**Figure 4:**
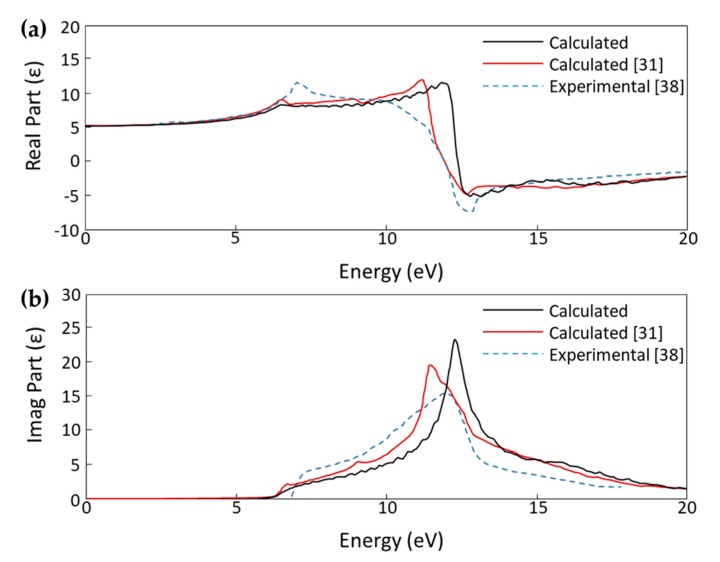
(**a**) Real part and (**b**) imaginary part of the calculated dielectric constant (ε) plots for bulk diamond comparing our calculated results with calculated results by Xiang et al. and experimental results by Philipp and Taft [31,38].

We calculated the optical spectrum of bulk diamond and plotted the dielectric constant to compare our results to a recent theoretical study by Xiang et al and experimental data published by Philipp and Taft to further validate the reliability of our calculation parameters [31,38]. The comparison plots for the real and imaginary parts of the dielectric constant in Figure 4 shows that our DFT method produces results that are in good agreement with other published theoretical work and experimental data.

After the H-diamond and MoO_3_ units had been fully geometrically optimised, they were interfaced with a strain of <1% being placed on the MoO_3_. To geometrically optimise the interface separation, the H-diamond atoms were fixed, while the MoO_3_ atoms were kept rigid, allowing for the oxide to move as one unit in the x, y, and z directions. The lattice parameters for the super cell were a = 60.861 Å, b = 7.581 Å, and c = 30.962 with a vacuum of 19 Å. 

**Figure 5:**
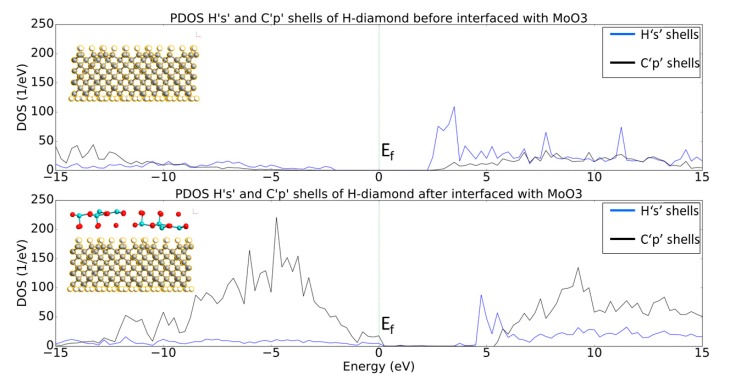
Projected Density of States (PDOS) plots of Hs and Cs shells before and after the H-diamond is interfaced with the oxide. The images inset are two-dimensional (2D) cuts in the middle of the systems just to show what atoms are being projected in the PDOS plots.

Figure 5 shows the projected Density of States (PDOS) for the ‘1s’ electron shells of the hydrogen and the ‘2p’ electron shells of the carbon atoms before and after the H-diamond has been interfaced with MoO_3_. From the top plot of Figure 5 is clear that, the pure bulk H-diamond still has a band gap of around ~5.5 eV.

The PDOS diagram reveals that, when the H-diamond is in contact with the MoO_3_, there is a shift of the PDOS to higher energies and the Fermi Level (E_F_) is in contact with the valence states. Moreover, the band gap of the diamond remains constant, but states of the Valance Band Maximum (VBM) have crossed the Fermi Level, which in turn means that charge transfer has occurred, as previously occupied states within the H-diamond are now vacant. This demonstrates that the electrons transferred from the diamond surface to the MoO_3_ layer, leaving the hole accumulation layers, as expected from the STD model. Or, in other words, the shift of the E_F_ from the middle of the bandgap toward the VBM also indicates p-type doping of the H-diamond. This is consistent with the recent experimental and simulation results that were published by other groups [27]. 

**Figure 6:**
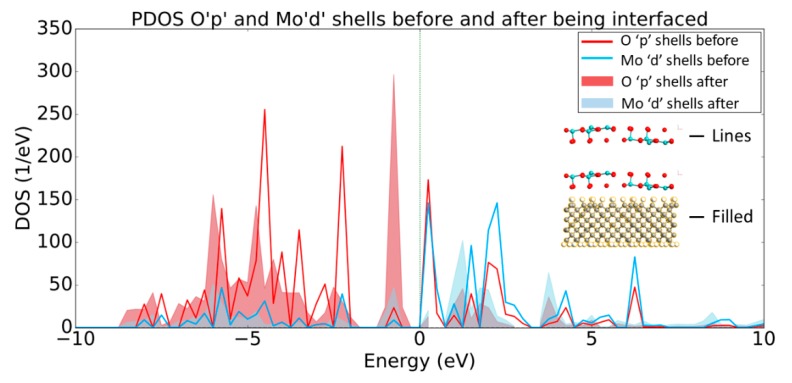
PDOS plot of O_p_ and Mo_d_ shells before (lines) and after (filled) being interfaced with the H-diamond. Inset are two-dimensional (2D) crops of the systems being shown in the PDOS, showing that the line and filled plots denote the MoO_3_ before and after being interfaced, respectfully.

The PDOS plot presented in Figure 6 shows the opposite trend regarding the band structure in comparison to the PDOS data for the diamond, which is consistent with the discussion in the previous paragraph. More specifically, the Mo_d_ and O_p_ shells have increased states that lie below the Fermi level after the MoO_3_ has been interfaced with the H-diamond, indicating that there has been charge transfer at the interface. Indeed, this charge transfer corresponds to an acceptance of electrons by the metal oxide from the diamond substrate.

**Figure 7:**
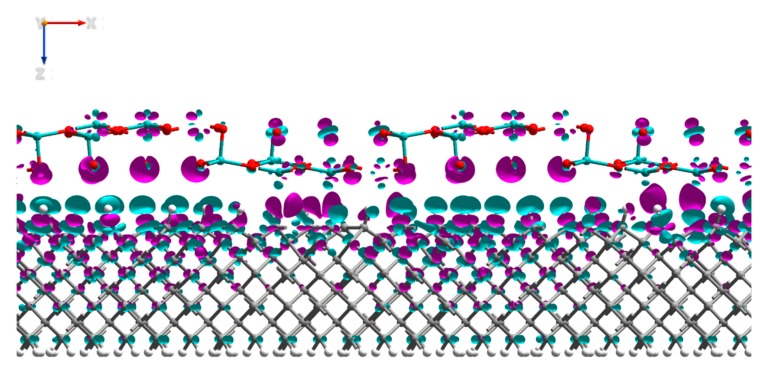
Side view of the MoO_3_:H-diamond interface structure showing the electron density differences. The purple regions represent electron accumulation and the green regions represent electron depletion (hole accumulation). The isosurface values are ±0.002 Bohr^−3^.

Additional to the PDOS data, we have analyzed the Mulliken charges and electron density differences of the interfaced system. The analysis of the Mulliken electron population of the system presented in Table 2 showed that electron density had transferred from the first four layers of the H-diamond to the MoO_3_, with negligible change to the electron density of the atoms deeper within the diamond. Consistent with the results that are presented in Table 2 is the image in Figure 7, which shows the electron density change for the MoO_3_:H-diamond interface. The green regions show where there is a loss of electron density and thus the accumulations of holes, and the purple regions show where there is an increase in electron density. The electron density difference isosurface shows that there is a hole accumulation that occurs near the surface of the diamond, while most of the electron density gained by the MoO_3_ clearly migrates to the oxygen atoms. Figure 7 and Table 2 are clear representations that the H-diamond loses electrons from the layers closest to the surface, showing that a 2DHG created by surface transfer doping has been formed in the H-diamond. Most of the charge transferred to the MoO_3_ migrates to the oxygen atoms, rather than the molybdenum atoms with a total transfer of 3.6 electrons from the H-diamond to the MoO_3_. Moreover, these results are in very good agreement with work by K. Xing et al., where they also demonstrate electron transfer from the H-diamond to the oxide [27]. 

Of the total electron density transferred to the oxide film, the oxygen atoms gain 2.1 electrons and the molybdenum atoms gain 1.5 electrons. The carrier concentration of the H-diamond would be ~6 × 10^13^ cm^−2^, given that the surface area of the system was ~60 Å × 10 Å, calculating the charge transfer per cm^2^. This is within the range of previously reported experimental carrier concentrations for MoO_3_ that was deposited on the surface of H-diamond substrates of 4 × 10^12^–1 × 10^14^ cm^−2^ [20,21,22,25,26]. Additionally, it is in very good agreement with the extracted from in-situ four-probe measurements hole density value of 2.7 × 10^13^ cm^−2^ and calculated charge transfer per unit cell of 4.7 × 10^13^ cm^−2^ for a monolayer coverage of MoO_3,_ reported by K. Xing et al [27]. 

Hence, to summarise, our simulations and analysis reveal that the MoO_3_ attracts electrons from the hydrogen terminated diamond in the interfaced system and there is charge transfer between the diamond and the oxide and, as a result, the diamond is p-type and the oxide is n-type doped.

### 3.2. V_2_O_5_: H-Diamond Interface

In order to further verify our findings regarding charge transfer to MoO_3_, we have performed simulations of another oxygen rich oxide experimentally shown to produce STD in H-diamond—V_2_O_5_ [13,23,24,25,26]. We utilized the same H-diamond cell used for the H-diamond:MoO_3_ system and interfaced it with a fully relaxed V_2_O_5_ unit cell, placing a strain of 1.02% on the V_2_O_5_ to create the H-diamond:V_2_O_5_ interface. The super cell lattice parameters were a = 39.231 Å, b = 12.636 Å, c = 50, with a vacuum of 36 Å. The interface was optimised by the same process that was used for the H-diamond:MoO_3_ interfacial distance and position.

**Figure 8:**
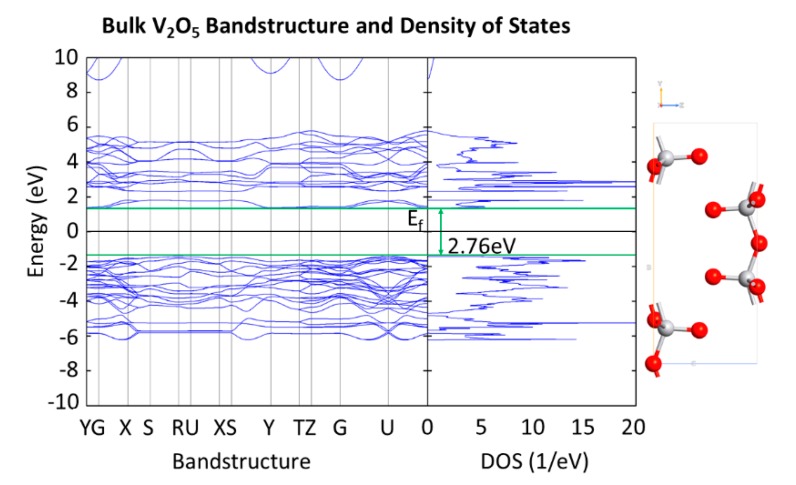
Bandstructure and DOS of bulk V_2_O_5_ showing a band gap of 2.76 eV.

Figure 8 depicts the bandstructure and DOS calculated for bulk V_2_O_5_. The band gap calculated is 2.76 eV, which is comparable to the optical bandgap of 2.8 eV reported for 100 nm thick thermally evaporated V_2_O_5_ films [39].

**Figure 9:**
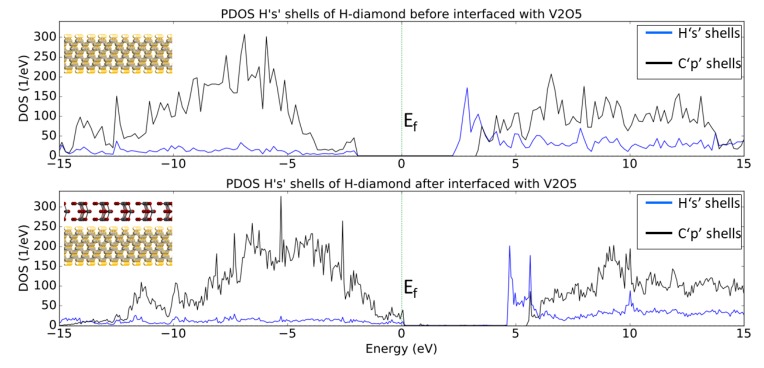
PDOS plots of Hs and Cs shells before and after the H-diamond is interfaced with V_2_O_5_. The images inset are 2D cuts in the middle of the systems just to show what atoms are being projected in the PDOS plots.

Figure 9 shows the PDOS for the ‘1s’ electron shells of the hydrogen and the ‘2p’ electron shells of the carbon atoms before and after the H-diamond has been interfaced with V_2_O_5_. When H-diamond is interfaced with V_2_O_5_ there is a shift of the PDOS of the diamond to higher energies with some states now crossing the Fermi Level (E_F_). Indeed, this is very similar to the observed trend when the H-diamond is interfaced with MoO_3_, where the VBM is in contact with the E_F_. This suggests that the diamond has lost electrons as states in the diamond that were occupied before being interfaced, are now vacant. The gap between the VBM and CBM remains the same as the diamond’s bulk band gap value. Additionally, Figure 9 reveals a similar trend regarding the DOS movement to the results that are shown in Figure 5. Indeed, the E_F_ has moved closer to VBM, which indicated that there has been charge transfer at the interface and that the H-diamond has been p-type doped. 

**Figure 10:**
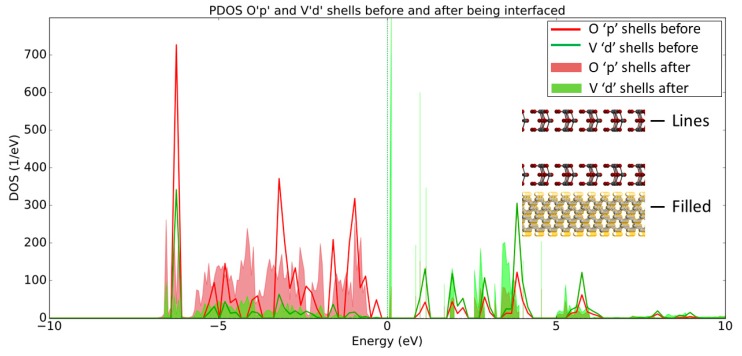
PDOS plot of O_p_ and V_d_ shells before (lines) and after (filled) being interfaced with the H-diamond. Inset are 2D crops of the systems being shown in the PDOS, showing that the line and filled plots denote the MoO_3_ before and after being interfaced, respectfully.

The PDOS presented in Figure 10 shows that the O_p_ and V_d_ shells have increased states that lie below E_F_ after being interfaced with the H-diamond, much in the same way that the O_p_ and Mo_d_ shells did. This result suggests that the Vanadium Pentoxide acts in a similar way to the Molybdenum Trioxide to create a 2DHG in the H-diamond when in direct contact with the metal oxide.

**Figure 11:**
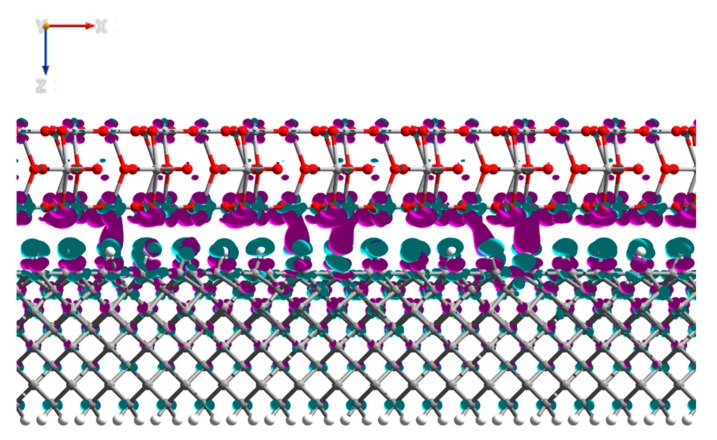
Side view of the V_2_O_5_:H-diamond interface structure showing the electron density differences. The purple regions represent electron accumulation and the green regions represent electron depletion (hole accumulation). The isosurface values are ±0.0025 Bohr^−3^.

When the Mulliken electron population (Table 3) of the interfaced system was compared to the charges of the H-diamond and V_2_O_5_ individually, it showed that there was a charge transfer of 5.1 electrons from the top four layers of the H-diamond to the oxide, again with negligible transfer propagating from deeper within the H-diamond (Figure 11), showing a 2DHG, as observed with the MoO_3_.

Similar to the MoO_3_, most of the charge transferred migrated to the O atoms in the V_2_O_5_ as opposed to the V atoms, with an electron density gain of 4.9 and 0.2 for the O and V atoms, correspondingly, see Table 3. Although there was slightly more charge transfer than was observed for MoO_3_, the supercell for the V_2_O_5_:H-diamond system is slightly larger. This meant, when considering the surface area of the system, the calculated carrier concentration per cm^2^ created by the V_2_O_5_ was 2.17 × 10^13^ cm^−2^, which, in turn, is slightly less than the carrier concentration seen with MoO_3_. The experimental carrier concentration of H-diamond with a thin film of V_2_O_5_ thermally deposited on the surface, by means of Hall measurements, is measured to be 1.8 × 10^13^ cm^−2^ [13]. Previous experimental result on H-diamond:V_2_O_5_ of reported values in the range of 1.8 × 10^13^ cm^−2^–1.1 × 10^14^ cm^−2^, agree with the carrier concentration we have obtained from our DFT simulations [23,24,25,26]. Hence, our results demonstrate that V_2_O_5_ acts as highly efficient surface acceptor for modulating the carrier concentration and, hence, surface conductivity of the hole conducting channel on diamond. 

### 3.3. MoO_3_ and V_2_O_5_ Comparison

**Figure 12:**
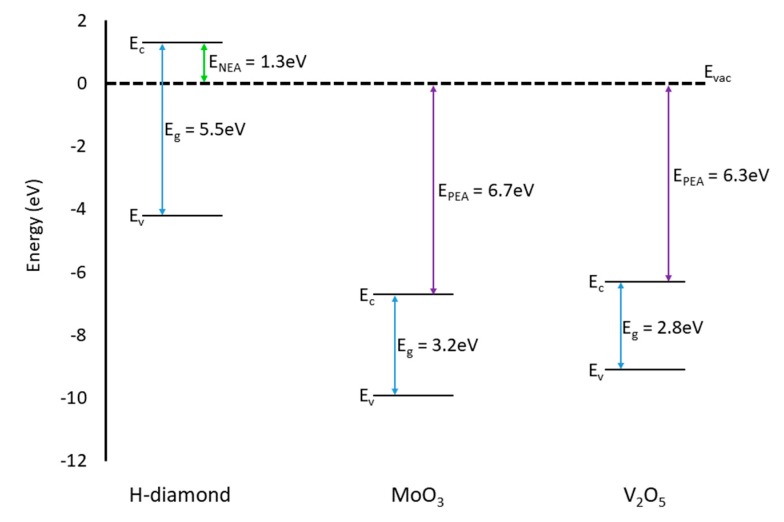
Energy band diagram showing the VBM and CBM positions for H-diamond (left hand side), MoO_3_ (middle), and V_2_O_5_ (right hand side) [30,40,41].

Figure 12 presents an energy band diagram for H-diamond, MoO_3_, and V_2_O_5_ materials relative to the vacuum level (E_VAC_). In terms of the relation of the VBM in diamond and CBM positions of the oxides both oxides should interact similarly with the diamond substrate. For an adsorbate to accept electrons and, therefore, inject holes into the H-diamond it must have a CBM that lies below the VBM of the H-diamond. The CBM of both oxides are at approximately the same energy level below the VBM of the diamond and, therefore, it is expected that both oxides will yield similar results when they are interfaced with H-diamond.

To evaluate the interaction between the H-diamond and the oxides, adsorption energies for both oxides were calculated using the following formula:

(1) $$E_{adsorption}=E_{H-diamonds/oxide}-E_{H-diamond}-E_{oxide}$$

*E_H-diamond/oxide_* is the total energy of the interfaced H-diamond and oxide system. *E_H-diamond_* and *E_oxide_* are the energies of the individual components of the interfaced system. Therefore, the adsorption energy is the difference in energy between the whole interfaced system and each individual component: H-diamond and the oxide. The adsorption energies for both oxides, as listed in Table 4, were negative values, which indicate that both processes are driven by a favorable exothermic reaction. Hence, both oxides can be physically absorbed (physisorption) on the diamond surface. We believe that the process is physisorption instead of chemical absorption, because the only one 1s H electrons and the free 2p electron from the C atoms from the diamond surface form a stable covalent bond. Hence, there is no free electron for the H atom to create a chemical bond with either the transition metal or the oxygen from the metal oxide.

However, it can also be said that the adsorption energy is not responsible for increased charge transfer, given that the adsorption energy of the MoO_3_ is more than two times weaker than the V_2_O_5_, yet promotes greater charge transfer at the interface. It appears that the most important factor in the amount of charge transfer between H-diamond and the two oxides is the amount of O atoms present in the metal oxide, since, in both cases, this is where the majority of the electron density transferred at the interface migrates. Hence, our result would indicate that, in order to improve the charge transfer and, in turn, increase the carrier concentration in the H-diamond, it would be advantageous to use oxides that have a higher oxygen concentration. 

Moreover, metal oxide films are usually deposited onto H-diamond in a vacuum, which can lead to the formation of oxygen deficient amorphous oxide films. Therefore, we hypothesise that depositing the oxides in an oxygen rich atmosphere or using oxides that have a higher oxygen concentration could lead to increased charge transfer and enhanced STD. 

## 4. Discussion

In this work, we investigated models for the surface transfer doping effect induced in hydrogen-terminated diamond when interfaced with MoO_3_ and V_2_O_5_. We simulated the interfaces of the hydrogen-terminated (100) diamond surface with (100) MoO_3_ and (100) V_2_O_5_ while using DFT calculations. The PDOS data show there is a shift of the VBM and CBM bands when the H-diamond is interfaced and, thus, a transfer of electrons from the H-diamond to the metal oxides. MoO_3_ and V_2_O_5_ are both found to readily act as electron acceptors and create a 2DHG in the H-diamond. The carrier concentration in the H-diamond has been calculated using DFT simulations to be 6 × 10^13^ cm^−2^ and 2.17 × 10^13^ cm^−2^ for MoO_3_ and V_2_O_5_, respectfully, which are similar to the previously reported experimental values. These values are in good agreement with the range of experimental and simulation studies reported thus far, but underestimate the highest 2DHG carrier concentration values reported to date for these systems. Further refinement of these models to account for variation in surface morphology and hydrogen/oxygen coverage of the diamond surface as well as crystallinity/stoichiometry of the oxide layers will allow for finer optimisation of these models and provide a deeper understanding of the complex charge transport mechanisms at the hydrogen-terminated diamond surface. 

However, from these simulations, it is clear that the oxygen atoms play a major role in the amount of charge transfer from the hydrogenated diamond to the metal oxide layers as the majority of the electron density transferred from the diamond migrates to the oxygen atoms. These results suggest that either depositing the metal oxide layers in an oxygen-rich atmosphere to reduce oxygen deficient oxide layers, or the investigation of new acceptor adsorbates with higher oxygen concentrations could lead to improved surface transfer doping in diamond. Moreover, our simulations show that, after depositing MoO_3_ and V_2_O_5_ on the H-diamond surface, the metal oxides show metallic behaviors due to the fact that the Fermi level is inside of the valence band. This observation is consistent with very recent experimental work that was conducted at low temperature, which proves that that MoO_3_/V_2_O_5_ doped H-diamond systems will show a metallic behavior rather than carrier freeze out at cryogenic temperature regime [42]. Hence, this allows for the observation of some exotic quantum transport phenomena, such as phase-coherent backscattering. 

## Figures and Tables

**Table 1:** Bandgaps produced for bulk diamond by different functionals using Generalised Gradient Approximation (GGA)-Half exchange correlation. Perdew, Burke, and Ernzerhof (PBE) was chosen, because it agreed with the experimental value of 5.5 eV.

Functional	Bulk Diamond Bandgap (eV)
BLYP	5.83
BP86	5.68
BPW91	5.63
PBES	5.42
PW91	5.62
RPBE	5.64
XLYP	5.86
PBE	5.59

**Table 2:** The calculated Mulliken charges for the MoO3-doped diamond surface.

Material	Before Adsorption	After Adsorption	Change in Mulliken Charge
H-Diamond	1680.0	1676.4	−3.6
MoO_3_	767.9	771.5	3.6
Mo	286.7	288.2	1.5
O	481.2	483.3	2.1
Surface H Layer	48.4	38.2	−10.2
First Carbon Layer	198.9	203.0	4.1
Second Carbon Layer	188.7	190.8	2.1
Third Carbon Layer	189.8	190.3	0.5
Bottom H Layer	95.5	95.0	−0.5

**Table 3:** The calculated Mulliken charges for the MoO3-doped diamond surface.

Material	Before Adsorption	After Adsorption	Change in Mulliken Charge
H-Diamond	2519.9	2514.8	−5.1
V_2_O_5_	1343.6	1348.7	5.1
V	520.4	520.6	0.2
O	823.2	828.1	4.9
Surface H Layer	72.4	55.2	−17.2
First Carbon Layer	298.4	305.4	7.0
Second Carbon Layer	283.0	287.1	4.1
Third Carbon Layer	284.7	285.8	1.1
Bottom H Layer	143.3	142.7	−0.6

**Table 4:** Adsorption energies corresponding to the two different oxides on H-diamond.

Adsorbate	Adsorption Energy (eV)
MoO_3_	−2.94
V_2_O_5_	−6.41
